# Immune phenotype is differentially affected by changing the type of bovine respiratory disease vaccine administered at revaccination in beef heifers

**DOI:** 10.3389/fvets.2023.1161902

**Published:** 2023-04-17

**Authors:** Cassidy Reddout, Lily P. Hernandez, Christopher C. L. Chase, Paul Beck, Frank White, Janeen L. Salak-Johnson

**Affiliations:** ^1^Department of Animal and Food Sciences, Oklahoma State University, Stillwater, OK, United States; ^2^Department of Veterinary and Biomedical Sciences, South Dakota State University, Brookings, SD, United States; ^3^Elanco Animal Health, Greenfield, IN, United States

**Keywords:** bovine respiratory disease, cattle, cytokines, immunoglobulins, serum-neutralizing titers, vaccine

## Abstract

During preconditioning, modified-live vaccines are frequently administered to beef calves before weaning. In this study, we began to characterize the immune phenotype of calves that received a modified-live vaccination at 3–4 months of age and then either received the same modified-live or an inactivated vaccine upon arrival at the feedlot (weaning) and 28 days post-arrival (booster). Innate and adaptive immune measures were assessed before revaccination and 14 and 28 days post. Heifers that received three doses of the modified-live vaccine exhibited a relatively balanced immune response based on increases in mean cytokine concentrations (IL-17, IL-21) and total immunoglobulin-G (IgG) and subsets IgG1 and IgG2, which are related to both arms of the adaptive immune system. Conversely, heifers that received one dose of modified live and two doses of the inactivated vaccine had a more robust neutrophil chemotactic response and greater serum-neutralizing antibody titers, resulting in an enhanced innate immune and a skewed proinflammatory response. These results indicate that the revaccination protocol used after initial vaccination with a modified-live vaccine differentially influences the immune phenotype of beef calves, with three doses of modified live inducing potentially immune homeostasis and a combination of modified live and inactivated vaccines inducing a skewed immune phenotype. However, more research is needed to determine the protective efficacy of these vaccination protocols against disease.

## 1. Introduction

Bovine respiratory disease (BRD) is the leading cause of morbidity and mortality in cattle (1), despite years of vaccine development and research. This is partly due to the complexity of the pathogenesis involving viruses, bacteria, and the host immune response culminating in the BRD complex and causing disease (2). Since BRD is a multifactorial disease complex, both humoral (Th-2) and cell-mediated (Th-1) immune responses are necessary for viral clearance and protection (3, 4). Pathogenesis within BRD may be due in part to the ability of pathogens to induce a skewed immune response. Bovine Respiratory Syncytial Virus (BRSV), one of the viruses making up the BRD complex, induces a skewed humoral immune response and a depression in cell-mediated response (5). A similar trend can be seen in RSV, a human equivalent respiratory virus in which those with an imbalance of Th-1 and Th-2 associated factors develop the more severe disease (6). This indicates that a balance between humoral and cell-mediated may be optimal for protection and decreased disease severity. Using multiple immunologic indicators such as immunoglobulins, cytokines, and neutralizing antibodies can provide an overall immune profile indicative of a bias or balance. Furthermore, some immunological measures used to assess vaccine response, such as immunoglobulin G and serum-neutralizing antibody titers, may be correlated (7).

Serum-neutralizing antibody titers are most often used to assess the immune response to vaccines, but with contradictory outcomes. Some research shows that even low antibody titers protect against challenges (5), while others found that lower levels were associated with a more severe pathology (8). Previous studies showed that administering at least one MLV vaccine during the preconditioning period resulted in higher BRD-specific neutralizing antibody titers (9, 10). In contrast, others found that two doses of an inactivated vaccine resulted in higher titers for parainfluenza-3 and infectious bovine rhinotracheitis, two viruses within the BRD complex (11). The threshold of titer required for protection is unknown (5), and detectable humoral (Th-2) or cell-mediated (Th-1) immunity levels do not assure protection against infections. Therefore, multiple immunologic indicators, such as immunoglobulins and cytokines, should be analyzed in conjunction with neutralizing antibodies to more effectively determine the vaccine’s efficacy and ability to prevent disease.

Although BRD vaccines are most often used in feedlot cattle (1), the efficacy and protection provided are inconsistent. This may be partly attributed to differences in vaccination protocol, vaccine formulation, and whether exposure occurs naturally or through challenge studies (12). More specifically, the type of vaccine can be classified as inactivated/killed (INA) or modified-live (MLV). An inactivated vaccine contains killed cells or cell subunits of the specific pathogen(s), which cannot replicate, must be administered at higher doses, and require an adjuvant to induce an adequate immune response (13). While an MLV vaccine contains pathogens that can reproduce *in vivo* (13), infectivity is reduced due to being attenuated and should not cause disease (14). However, these vaccines often have greater immunogenicity since they mimic natural infection, leading to the induction of both humoral and cell-mediated immune responses (13). Moreover, MLV and INA vaccines can differentially drive different aspects of the immune system (13). Thus, determining vaccine efficacy and the immune phenotype induced by vaccination type and protocol is important in using vaccination strategies to manage BRD. By determining the immune phenotype induced by either modified-live or inactivated vaccination protocols, we can utilize the information to begin to determine which regime leads to a more protective immune response.

Characterizing the immune phenotype induced by either vaccination protocol will allow for speculation into which may result in a more protective combination of cell and antibody-mediated immune function and, thus, which may be more effective when administered. Therefore, this study aimed to characterize the immunological phenotype of a subset of heifer calves initially vaccinated with a BRD modified-live vaccine at 3–4 months, revaccinated with either an MLV or an INA vaccine at weaning, and then boosted 28 days later with the same vaccine they received at weaning by analyzing immune measures associated with innate and adaptive immune systems.

## 2. Materials and methods

All procedures were approved by the Oklahoma State University Institutional Animal Care and Use Committee (Protocol No. AG-15-21 and AG-19-8).

### 2.1. Animal management and experimental treatments

Before enrollment in the study, all animals were vaccinated at 3–4 months of age with a single dose of a modified-live vaccine (MLV; Titanium^®^ 5; Elanco Animal Health, Greenfield, IN, United States). This vaccine contained infectious bovine rhinotracheitis (IBR), bovine viral diarrhea virus (BVDV) type 1 and 2, parainfluenza 3 (PI3), and bovine respiratory syncytial virus (BRSV). Angus calves (initial BW = 233 ± 96 kg) were weaned and transported from the Oklahoma State University Field and Research Service Unit (Valliant, OK, United States) to Oklahoma State University Willard Sparks Beef Research Center (WSBRC; Stillwater, OK, United States). Upon arrival at WSBRC, calves were held overnight in a dry lot with *ad libitum* access to fresh water and prairie hay. The following day, all calves were administered a Clostridium chauvoei (Blackleg), septicum (Malignant edema), novyi (Black disease), sordellii and perfringens Types C and D (Enterotoxemia), and Moraxella bovis (Pinkeye, or infectious bovine keratoconjunctivitis) vaccine (20/20 Vision^®^ with SPUR^®^, Merck Animal Health, Madison, NJ, United States), Mannheimia haemolytica (NUPLURA™ PH; Elanco Animal Health), and an oral anthelmintic (Safeguard; Merck Animal Health) according to the manufacturer’s label. Then, calves were assigned to mixed-sex pens balanced by sex and weight. It should be noted that all utilized animals’ vaccination and medical history was known. Pens were randomly assigned to one of two revaccination treatments: modified-live virus vaccine (MLV-Titanium^®^ 5) or inactivated virus vaccine (INA). The INA vaccine contained inactivated viruses IBR, BVDV 1 and 2, PI3, and BRSV (ViraShield^®^ 6; Elanco Animal Health). Before moving animal groups to their assigned treatment pen, blood samples were taken, and calves were administered their vaccine treatment. Then 28 days later, they received a booster of the same vaccine treatment. This resulted in two treatment groups: **MLV3** (MLV = 3 doses) and **MLV + INA2** (MLV = 1 dose; INA = 2 doses).

In order to further characterize the immune phenotype, a subpopulation of heifers (*n* = 28) was selected from six treatment pens (*n* = 3 MLV3; *n* = 3 MLV + INA2) balanced for body weight. Blood samples were obtained 24-h after arrival at WSBRC and prior to revaccination treatment (d 0) and at 14 and 28 days-post revaccinations (**PRv**) and post-booster (**PB**). Animals were also weighed on blood collection days. Only data from this subpopulation are presented.

### 2.2. Sample collection and processing

Whole blood and serum samples were collected *via* jugular venipuncture using serum, heparin (neutrophils and plasma), and EDTA (complete cell counts; CBC) vacutainers (BD Vacutainers; Franklin Lakes, NJ, United States). The CBC was determined electronically from whole blood using the Element HT5 Hematology Analyzer (Heska, Loveland, CO, United States). Serum tubes were allowed to clot at room temperature, centrifuged at 3,000 × g for 20-min, and then aliquoted. Plasma samples were stored at −20°C and serum at −80°C until subsequent analysis.

### 2.3. Neutrophil isolation and chemotaxis

Whole blood was transferred to a 15 mL sterile conical tube and centrifuged at 1,800 rpm for 30-min at room temperature. Following centrifugation, the buffy coat and 25% of the packed red blood cell (RBC) layer were discarded. The remaining cell content was transferred to a 50 mL conical tube, lysed using cold, sterile deionized water, and then returned to an isotonic solution by adding 10X phosphate-buffered saline (PBS: Sigma, St. Louis, MO, United States). Tubes were centrifuged at 500 × g for 15-min at 4°C. The supernatant was removed, and the cell pellet was washed in Roswell Park Memorial Institute (RPMI) 1,640 media (Gibco, Waltham, MA). Then, the cell pellet was re-suspended RPMI and placed on ice until used in the assay.

The ability of neutrophils to randomly (media; control) or directly migrate (chemotaxis) toward chemoattractant recombinant human complement-5a (C5a, 10^−7^ M; Bio-vision, Waltham, MA, United States) was assessed following procedures previously described by Salak et al. (15) and modified by Auchtung et al. (16). Neutrophils were adjusted to 3 × 10^6^ cells/mL. In duplicate, the chemoattractants were added to the bottom chamber and the cells to the top. The two chambers were separated by a polycarbonate filter (pore size 5 μm; Neuro Probe, Cabin John, MD, United States). The chamber was incubated for 45-min at 37°C in a humidified incubator (5% CO^2^). The filter was fixed and stained using the Hema-3 Stain system (Fisher Scientific, Waltham, MA, United States). Cells that migrated to the underside of the filter were counted using a light microscope under oil emersion at 100×.

### 2.4. Cortisol, cytokines, and immunoglobulin-G subsets

Plasma cortisol was measured using a commercially available enzyme-linked immunoassay (ELISA) following the manufacturer’s protocol (Enzo Life Sciences, Farmingdale, NY, United States) with minor modifications. Samples were diluted 1:8 in assay buffer and run in duplicate in a 96-well microtiter plate coated with goat anti-mouse IgG. The conjugate (alkaline phosphatase-conjugated with cortisol) and antibody (mouse monoclonal antibody to cortisol) were added. Plates were placed on a shaker at 500 rpm for 2 h at room temperature and washed three times, then substrate (P-nitrophenylphosphate) was added, and plates were incubated for 1 h at room temperature. The reaction was stopped with a solution provided. Plates were read using a microplate reader (BioTek Epoch, Winooski, VT, United States) at 405 nm. A standard curve was used to determine the concentration of the unknown samples using the Gen5 Data Analysis Software (BioTek). The minimal detectable concentration of the assay was 56.7 pg./mL.

Interleukin-4, −6, −10, −17, −21 (Invitrogen Corp., Waltham, MA), and − 8 (Biomatik, Ontario, CA) were measured using commercially available bovine ELISA kits for each cytokine following the manufacturer’s protocols. Briefly, the 96-well microtiter plates were pre-coated for all cytokines except for interleukin-6 and -8. Standards and samples were pipetted in duplicate, and plates were incubated at room temperature with gentle shaking. Anti-bovine detection antibodies IL-4 or IL-6 and biotin-conjugated antibodies IL-10, IL-17, or IL-21 were added to the appropriate plate and incubated for 1 h at room temperature. Plates were washed several times, Streptavidin-HRP was added to each well, and plates were incubated with moderate shaking at room temperature for 30 min (IL-6 and IL-4) or 45 min (IL-10, IL-17, and IL-21). Tetramethylbenzidine dihydrochloride (TMB) substrate was added to each well. Plates were incubated for 20 min (IL-6, IL-4) or 30 min with gentle shaking (IL-10, IL-17, IL-21); the reaction was terminated with stop solution, and plates were read at 450 nm wavelengths using the BioTek Epoch plate reader. Using Gen5 Data Analysis Software (BioTek, Winooski, VT, United States). The minimal detectable concentration of the assays for interleukin-4, −6, −8, −10, −17, and − 21 were 15.6 pg./mL, 78.1 pg./mL, 5.9 pg./mL, 0.12 ng/mL, 2 pg./mL, and 0.41 ng/mL, respectively.

Immunoglobulin-G (IgG) subsets IgG1, and IgG2 concentrations were measured at South Dakota State University (Brookings, SD, United States) using commercially available bovine IgG1 (E11-16) and IgG2 (E11-17) ELISA kits (Bethyl Laboratories Inc., Montgomery, TX, United States), following the manufacturer’s protocol. Briefly, samples were diluted in sample diluting buffer at 1:500,000, and samples and standards were pipetted in duplicate onto 96-well microtiter plates coated with either anti-bovine IgG1 or IgG2 antibody. Plates were incubated at room temperature for 1 h and washed four times. Anti-IgG1 or anti-IgG2 detection antibody was pipetted into the wells. Plates were incubated at room temperature for 1 h and washed four times. Horseradish peroxidase solution was added to each well, and plates were incubated at room temperature for 30 min and washed. The TMB substrate was added, and plates were incubated in the dark for 30-min at room temperature. The reaction was stopped using a provided solution, and plates were read at 450 nm. The standard curve was plotted using Soft-Max pro software (Molecular Devices LLC, CA, United States) to estimate concentrations of IgG1 and IgG2. Total IgG was calculated by adding the values of the concentration of IgG1 and IgG2. The minimal detectable concentration of both assays was 1.0 ng/mL.

### 2.5. Serum neutralizing antibody titers

Serum samples were shipped to Texas Veterinary Medical Diagnostic Laboratory (Canyon, TX, United States) on dry ice to analyze serum neutralizing (SN) antibody titers for infectious bovine rhinotracheitis (IBR), bovine viral diarrhea virus types 1a, 1b, and 2 (BVDV 1a, 1b, 2), parainfluenza 3 (PI3) and bovine respiratory syncytial virus (BRSV). The reciprocal of the highest dilution of serum that neutralizes the infectivity of the virus was determined as the SN antibody titer.

### 2.6. Statistical analysis

Data were analyzed using the mixed model with repeated measures and Pearson Correlation procedures in SAS 9.4 (Inst. Inc., Cary, NC, United States). All traits were tested for departure from a normal distribution; therefore, Log-transformation was applied to cytokines and serum-neutralizing antibody titers. The model included fixed effects of vaccination treatment (MLV or INA). Significance was set at (*p* ≤ 0.05), but trends were discussed at (*p* > 0.05) to (*p* ≤ 0.10).

## 3. Results

### 3.1. Interactive treatment by day effects

#### 3.1.1. Leukocyte populations and hematology

**Figure 1:**
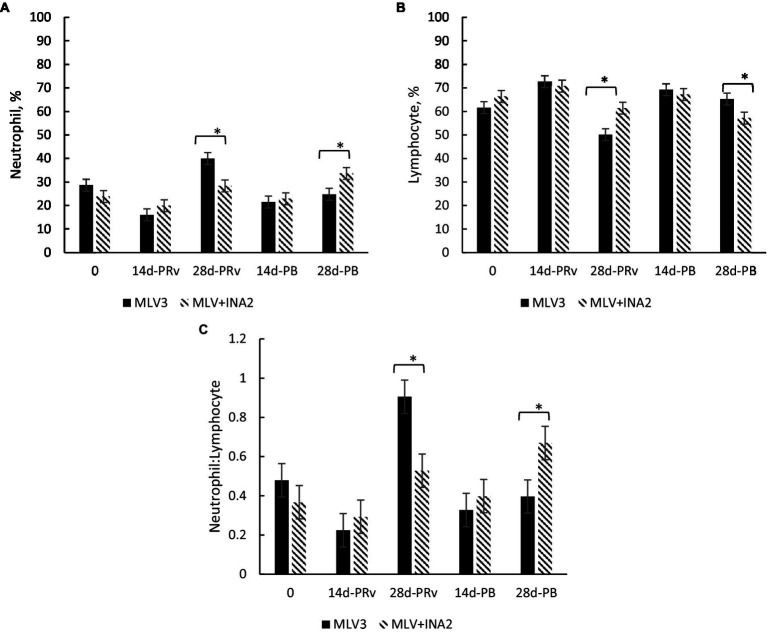
Effect of vaccine treatment on percentage of neutrophils **(A)**, percentage of lymphocytes **(B)** and the ratio of neutrophils to lymphocytes **(C)** at 14 and 28 days post-revaccination (PRv) and post-booster (PB), *n* = 28 heifers. Data are expressed as means ± standard error of the mean. Means with * between treatments within a day differ at *p* < 0.05. Treatment × Day = *p* = 0.001 for Neutrophil %, *p* = 0.003 for Lymphocyte %, *p* = 0.003 for Neutrophil to Lymphocyte ratio.

A treatment × day interaction occurred for percentages of neutrophils (*p* = 0.005), lymphocytes (*p* < 0.05), and the neutrophil-to-lymphocyte ratio (N:L; *p* = 0.009) shown in Figure 1. At 28d-PRv, neutrophil percentages were higher among MLV3 heifers than MLV + INA2 (Figure 1A), while lymphocyte percentages were higher among MLV + INA2 heifers than MLV3 (Figure 1B). The shift toward increased neutrophils and decreased lymphocytes resulted in a 53% greater N:L ratio among MLV3 vaccinated heifers than the MLV+INA2 ones at 28d-Prv (Figure 1C). Interestingly, at 28d-PB, the opposite pattern occurred among treatment groups. The MLV + INA2 vaccinated heifers had higher percentages of neutrophils than MLV3 ones (Figure 1A), and the MLV3 vaccinated heifers had higher percentages of lymphocytes than the MLV + INA2 (Figure 1B). Moreover, at 28d-PB, the N:L ratio was 51% greater among MLV + INA2 vaccinated heifers than MLV3 (Figure 1C). All other leukocyte populations and blood parameters were similar across treatment days post-revaccination or booster (*p* > 0.20; treatment × day).

#### 3.1.2. Neutrophil chemotaxis and chemokinesis

A treatment × day interaction occurred for neutrophil chemotaxis (*p* < 0.0001) and chemokinesis (random; *p* = 0.0004). Specifically, at 14d-PRv, chemotaxis was 29% higher among MLV + INA2 vaccinated heifers (501 ± 19.3, No.) than the MLV3 treatment group (374 ± 19.3, No.; *p* < 0.0001). However, by 28d-PRv, chemotaxis was similar between treatment groups (*p* = 0.68). Conversely, at 14d-PB, neutrophil chemotaxis was 63% higher among MLV3 vaccinated heifers (93.5 ± 11.7, No.) than the MLV + INA2 treatment group (48.7 ± 11.7, No.; *p* = 0.01). While chemokinesis only differed at 14d-PRv between treatment groups, with chemokinesis being 71% higher among the MLV + INA2 vaccinated heifers (382 ± 28.1, No.) than MLV3 ones (183 ± 28.1, No.; *p* < 0.0001). Overall, neutrophil chemotaxis and chemokinesis were 14 and 57%, respectively, among the heifers receiving one dose of MLV and two doses of INA than the heifers vaccinated with three doses of MLV.

#### 3.1.3. Serum neutralizing antibody titers

**Figure 2:**
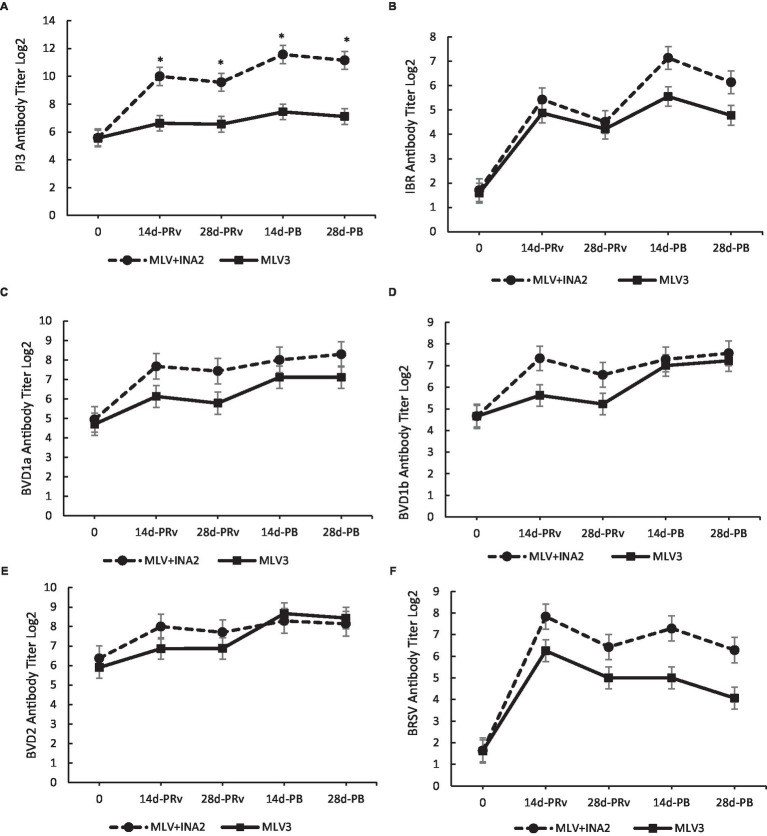
Effect of vaccine treatment by day on serum neutralization antibody titers for Parainfluenza 3 **(A)** at 14 and 28 days post-revaccination (PRv) and post-booster (PB) Treatment × Day interactive = *p* < 0.0001. No effect of vaccine treatment by day was found for serum neutralizing antibody titers for Infectious Bovine Rhinotracheitis **(B)**, Bovine Viral Diarrhea Virus type 1a **(C)**, 1b **(D)** or 2 **(E)**, or Bovine Respiratory Syncytial Virus **(F)**. Data are expressed as means ± standard error of the mean. Means between treatments within a day with * differ at *p* < 0.05.

Interestingly, the only treatment × day interaction that occurred for serum-neutralizing titers was for PI3. The MLV + INA2 vaccinated heifers had higher serum neutralization values at 14 and 28 days PRv and PB than the MLV3 vaccinated heifers (*p* < 0.0001; Figure 2A). The highest titers occurred at 14d-PB. Overall, the MLV + INA2 calves produced higher amounts of serum-neutralizing PI3-specific antibodies post-vaccination, and their titers remained elevated throughout the entire experimental period. However, it should be noted that positive PI3 serum-neutralizing antibodies were measured among the MLV3 vaccinated heifers, but titers did not reach the magnitude of the MLV + INA2 group. Also, no significant treatment × day interactions existed for IBR, BVDV 1a, BVDV 1b, BVDV 2, or BRSV serum-neutralizing titers (Figures 2B–F). However, the antibody-specific titer patterns for these antigens were similar between treatment groups, with the MLV + INA2 vaccinated heifers always having the highest titers at all-time points, indicating that while the MLV3 did not reach the same magnitude, they still exhibited a similar change in titers values in response to revaccination and booster vaccination.

#### 3.1.4. Immunoglobulin G and subsets

**Figure 3:**
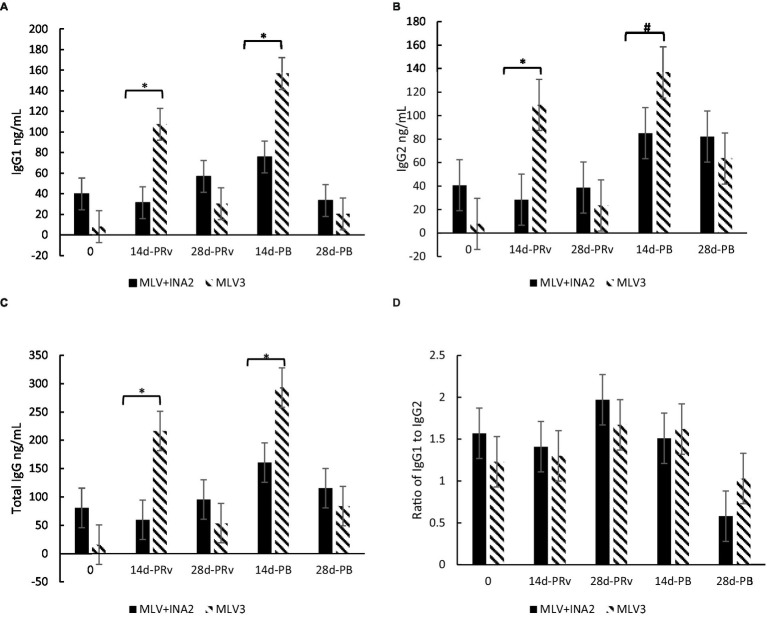
Effect of vaccine treatment by day on serum concentrations (ng/mL) of immunoglobulin G1 **(A)**, immunoglobulins G2 **(B)**, and total immunoglobulin G **(C)** and the ratio of Immunoglobulin G1:Immunoglobulin G2 ratio **(D)** at 14 and 28 days post-revaccination (PRv) and post-booster (PB), *n* = 28 heifers. Data are expressed as means ± standard error of the mean. Means with * between treatments within a day differ at *p* < 0.05 and means with # differ at *p* < 0.10. Treatment × Day = *p* < 0.0001 for IgG1, *p* < 0.05 for IgG2, *p* = 0.002 for Total IgG, and *p* > 0.65 for the Ratio of IgG1 to IgG2.

A treatment × day interaction occurred for IgG1 (*p* < 0.0001), IgG2 (*p* < 0.05), and total IgG (*p* = 0.002), but not for the IgG1:IgG2 ratio (*p* > 0.65). At 14d-PRv and 14d-PB, IgG1 concentrations were 110 and 70%, and IgG2 were 117 and 51% higher, respectively, among heifers receiving three doses of MLV than those who received one dose of MLV and two doses of INA (Figures 3A,B), resulting in greater total IgG levels among the MLV3 vaccinated heifers (Figure 3C). It is important to note that the highest IgG1 and IgG2 concentrations among the MLV3 treatment group occurred at 14d-PRv and 14d-PB compared to all other sample days. More specifically, IgG1 increased by 1,310% from day 0 to 14d-PRv in the MLV3 vaccinated heifers and 255% from 28d-PRv to 14d-PB. Also, IgG2 increased by 1,108% from day 0 to 14d-PRv and 500% from 28d-PRv to 14d-PB in these heifers. Conversely, IgG1 and IgG2 concentrations were not different across time points among the MLV + INA2 vaccination heifers. Although differences were detected between treatments across days for total IgG and subsets, the IgG1:IgG2 ratio was not different. All ratios were > 1 regardless of treatment, except at 28d-PB, the IgG1:IgG2 ratio was < 1 among the MLV + INA2 treatment group (Figure 3D).

### 3.2. Main effect of treatment

#### 3.2.1. Cortisol and cytokines

Mean plasma cortisol concentrations were higher for the MLV3 vaccinated heifers than MLV + INA2 ones (1,162 vs. 970 ng/mL, SE = 69.5; *p* = 0.05). These heifers also had higher mean concentrations of IL-17 (*p* = 0.012) and IL-21 (*p* < 0.05) but tended to have lower IL-8 (*p* = 0.10) and IL-6 (*p* < 0.10) than the MLV + INA2 treatment group (Table 1). Neither IL-4 nor IL-10 was affected by treatment (*p* > 0.20). These results may indicate the ability of the MLV3 group to induce both cell and antibody-mediated immune responses based on their cytokine profile.

**Table 1:** Effects of vaccine treatment on mean cytokine concentrations.^1,2^

Measure	MLV + INA2	MLV3	SEM	*p*-values
Interleukin-4, pg./mL	166.1	194.0	33.9	0.56
Interleukin-6, pg./mL	1097.1	589.1	207.2	<0.10
Interleukin-8 pg./ml	55.4	36.0	14.5	0.10
Interleukin-10, ng/mL	15.0	21.7	3.70	0.59
Interleukin-17, pg./mL	11.2	40.3	9.52	0.012
Interleukin-21, ng/mL	20.1	37.1	9.98	<0.05

^1^Data were log-transformed, but means are non-transformed ± standard error of the mean. ^2^Vaccine treatments were MLV + INA = 1 dose of modified-live, 2 doses of inactivated vaccine, and MLV = 3 doses of modified-live vaccine, *n* = 28 heifers.

#### 3.2.2. Serum neutralizing antibody titers

Overall, the MLV + INA2 group mean serum-neutralizing titers for IBR (*p* = 0.012), both BVDV1 subsets (*p* < 0.05), and BRSV (*p* < 0.001) were higher than the MLV3 treatment group (Table 2). These heifers also tended to have higher overall titers for BVDV2 (*p* = 0.06) than the MLV3 ones. It is important to note that both groups displayed positive titer values, indicating that both did produce antibodies specific to the viruses contained within the given vaccinations.

**Table 2:** Main effects of vaccine treatment on mean serum neutralizing antibody titers.^1,2,3^

Measure	MLV + INA2	MLV3	SEM	*p*-values
IBR	96.3	30.9	17.8	0.012
BVD1a	317.2	131.6	45.3	0.005
BVD1b	159.2	108.1	17.0	<0.05
BVD2	358.8	259.4	37.3	0.06
BRSV	184.2	61.7	24.6	<0.001

^1^Data are shown as least-squares means ± standard error of the mean (*n* = 28). ^2^Vaccine treatments were MLV + INA = 1 dose of modified-live, 2 doses of inactivated vaccine, and MLV = 3 doses of modified-live vaccine, *n* = 28 heifers. ^3^IBR, Infectious Bovine Rhinotracheitis; BVD1a, Bovine Viral Diarrhea type 1a; BVD1b, Bovine Viral Diarrhea type 1b; BVD2, Bovine Viral Diarrhea type 2; BRSV, Bovine Respiratory Syncytial Virus 4.

## 4. Discussion

Changing the type of vaccine administered at revaccination and a booster 28 day later in heifers that all received a modified-live vaccine at 3–4 months of age resulted in differing immunological phenotypes. Heifers revaccinated and boosted with a modified live vaccine (MLV3) displayed higher concentrations of both immunoglobulin G1 (IgG1) and immunoglobulin G2 (IgG2), which are indicative of a Th-2 and a Th-1 response, respectively, in response to both revaccination and booster. In contrast, heifers that received 1 dose of modified-live and 2 doses of inactivated vaccine (MLV + INA2) exhibited only an increase in IgG2 following the booster. The different cytokine profiles found between treatment groups further supported a more balanced immune response among those heifers receiving 3 MLV doses. In contrast, heifers that received 1 dose of modified-live and 2 doses of inactivated vaccine (MLV + INA2) had increased IgG2 following the booster, and heifers in the MLV + INA2 treatment group displayed a more robust serum-neutralizing antibody titer, often correlated with disease protection. These immune indicators imply that these animals’ immune status was skewed toward a Th-1 response.

A homeostatic balance between Th-1 and Th-2 responses often resolves the infection, whereas a skewed T-helper response is associated with more severe pathology (17). The corresponding Th-1 (cell-mediated or killing) antibody isotype IgG2 may aid in protection from pathological outcomes (18), and Th-2 (humoral or antibody) associated antibody, isotype IgG1, may be required for viral clearance (3), thus highlighting the importance of induction of both a Th-1 and Th-2 response. In the current study, those heifers in the MLV + INA2 treatment group had lower IgG1 and IgG2 concentrations at the sampling points post-revaccination and post-booster, which may indicate a delay in both humoral and cell-mediated immunity.

Despite the substantially greater magnitude of increase in IgG1 and IGg2 concentrations among the MLV3 vaccinated animals, there was no difference in the IgG1:IgG2 ratios between treatment groups. This ratio is one of the metrics used to differentiate between a Th-1 and Th-2 bias response. In this study, the Ig1:IgG2 ratio of the heifers in the MLV3 treatment group indicated a balanced Th-1 and Th-2 response since the ratio fell within the range of >0.5 but <2.0 (19). By the end of the study, the MLV + INA2 group had decreased to a level indicative of a bias toward a Th-1 bias, while the MLV3 group remained in the range indicative of a Th-1:Th-2 balance (19). The ratio changed following boosting, as the ratio for both groups was higher than the normal range for cattle prior to booster vaccination (7, 20). An interesting finding was that although the MLV + INA2 group had lower IgG concentrations, these heifers had higher serum-neutralizing antibody titers. This contrasted with Rajput et al. (7), who found a correlation between higher IgG levels and higher titer values following BVDV infection. Others also reported that serum-neutralizing antibody titers to BRSV and other viruses associated with the BRD complex are higher when calves are vaccinated with at least one modified-live vaccine (9, 10). Grooms and Coe (21) found that calves that received at least one MLV vaccine during preconditioning or weaning showed higher serum-neutralizing antibody titers for BVDV than calves that had only received an inactivated vaccine providing more evidence that the combined effect of the two vaccine types may lead to increased serum-neutralizing titer robustness. It must be noted that adjuvants used in the INA vaccine in Gooms and Coe was alum while INA vaccine in this study was water–oil emulsion. Notably, BVDV type 2 was the only neutralizing antibody not found to be significantly higher in the MLV + INA2 group. In another study, cattle were revaccinated with an INA vaccine after 2 previous MLV vaccines had significantly higher titers for BVDV 1 or 2 with almost no titer increases in animals given a third dose of MLV (22). This reduction in the magnitude of the SN titer may be due to differences in antigenic load since modified-live vaccines contain 2–3 logs less virus than INA as active virus replication following vaccine administration is necessary to achieve the immunogenic mass necessary for immune responsiveness (23, 24). The presence of active immunity from the initial vaccination may limit MLV replication, reducing antigenic mass and resulting in lower serum-neutralizing titer values, which was similar to findings reported by Walz et al. (22) and Royan (25). Another possibility is that a longer booster period would have increased titers. In another study, BVDV titers continued to increase for 2 months post-vaccination with an MLV vaccine (26), possibly indicating that the 56-day experimental period was not sufficient time to detect the peak neutralizing antibody titers in the MLV3 treatment group. However, the protective threshold titer level is unknown. A titer <1:4 is indicative of low protection, and others have found a poor correlation between vaccine efficacy and SN titers (27). In contrast, others have found that higher titer levels may be correlated with decreased disease severity. However, cell-mediated immunity is necessary for viral clearance (10), further highlighting the importance of a balanced Th-1 to Th-2 response.

Calves that received 3 MLV vaccinations had significantly higher mean plasma concentrations of IL-17 and IL-21, which are associated with Th-1 and Th-2, respectively, indicating that these elevated levels may be evidence of a more balanced Th-1:Th-2 immune profile among these calves in response to this vaccine treatment and protocol. Cytokines associated with Th-1 and Th-2 are upregulated during BRSV infection (28), and Th-1 cytokines have been associated with protection against BSRV pathology (4). Others have shown that calves vaccinated with the live-attenuated strain of BRSV mount a virus-specific IL-17 response (29). Further evidence for the importance of this balance is seen in severe RSV infection associated with an imbalance in the Th-1 and Th-2 cytokine ratio in human infants (6). Human RSV is similar to BRSV as both induce T-cell response biased toward a dysregulated Th-2 and Th-17-type cytokine response (30). Cells and fluid obtained from BRSV-infected calves were found to have higher concentrations of IL-4 and IL-13 (31), evidence of a similar cytokine response, with both cytokines being associated with a Th-2 response. However, we found no differences in IL-4 concentrations across days between treatment groups, thus implying that neither treatment group was skewed toward a Th-2 based on this cytokine, which drives a Th-2 response. However, the role of some of these cytokines, especially IL-17, in the immune response to the MLV vaccine and against intracellular pathogens is unclear, though IL-17 has been found to promote increased production of inflammatory IL-8, which is upregulated in the lungs of calves with BRSV (31). While the MLV + INA2 group did not exhibit a higher level of IL-17, they did tend to have higher overall IL-8 concentrations.

Neutrophils can be used as indicators of the innate immune response but also may play a role in cellular signaling for induction of the adaptive immune system (32), and peripheral neutrophils have been found to increase in response to BRD exposure (33). In the current study, peaks in neutrophil percentage occurred earlier in the experimental period in the heifers who received the MLV3 protocol. In contrast, the MLV + INA2 heifers exhibited a delayed onset of increased neutrophil percentage. This delay in neutrophil production in the MLV + INA2 vaccinated heifers may have reduced signaling for the production of adaptive immune response delaying the IgG2 response which is not seen until the end of the experiment. Another possible factor is cortisol which plays a role in the shift between Th-1 and Th-2-driven immune responses. Cortisol was found to be lower in the MLV + INA2 group overall. In low doses, it has been found to enhance the inflammatory response, and glucocorticoid receptor signaling has been speculated to sensitize pathways involved in innate immunity while suppressing those involved in adaptive (34). This may partly explain the trend for higher concentrations of IL-8 and earlier higher neutrophil function among the heifers who received an INA vaccine while failing to exhibit increases in markers of adaptive immunity until later in the experimental period. Overall, the MLV3 heifers exhibited higher concentrations of cytokines associated with a Th-1 and Th-2 response, furthering the speculation that heifers who receive three doses of a modified live vaccine may display a more balanced immune phenotype.

In summary, the immune response throughout the 56 day period differed by revaccination treatments. Heifers who received three doses of a modified-live vaccine for BRD had activation of both arms of the adaptive immune system, humoral and cell-mediated.They also exhibited marked immune responses through immunoglobulin G and its subtypes concerning the initial revaccination at weaning and then the booster 28 days later. This may indicate a more balanced immune response and potentially more immuno-protective phenotype. However, without presenting these cattle with an immune challenge, it is difficult to speculate if their immune phenotype is more effective at preventing or reducing disease severity. Despite increased overall titers, heifers who received one dose of a modified live and two doses of an inactivated BRD vaccine displayed a cell-mediated biased phenotype through overall IgG and cytokine responses. Heifers receiving the INA vaccine had a more delayed immune response than its MLV3 counterparts. This study indicates that revaccination protocol influences the immune phenotype. However, more research needs to be done to determine the protective value of these phenotypes.

## Data availability statement

The raw data supporting the conclusions of this article will be made available by the authors, without undue reservation.

## Ethics statement

All procedures were approved by the Oklahoma State University Institutional Animal Care and Use Committee (Protocol No. Ag-15-21 and Ag-19-8).

## Author contributions

JLS-J and PB: conceptualization and project administration. JLS-J: methodology, visualization and supervision. JLS-J, CC, and CR: formal analysis. CR and LH: investigation. JLS-J, PB, and CC: resources. JLS-J and CR: writing—original draft preparation. JLS-J, CC, CR, and FW: writing—review and editing. JLS-J, PB, and FW: funding acquisition. All authors have read and agreed to the published version of the manuscript.

## Funding

Elanco Animal Health partly financially supported this research, and the co-author FW was employed by Elanco Animal Health. JLS-J holds the Temple Grandin Endowed Chair, where laboratory resources for all research projects partly utilize these funds. This research was a contribution from the Oklahoma Agricultural Experiment Station, Stillwater, OK. The mention of trade names or commercial products in this article is solely to provide scientific information. It does not imply recommendation or endorsement by the Oklahoma Agricultural Experiment Station.

## Conflict of interest

FW is an employee by Elanco Animal Health.

The remaining authors declare that the research was conducted in the absence of any commercial or financial relationships that could be construed as a potential conflict of interest.

## Publisher’s note

All claims expressed in this article are solely those of the authors and do not necessarily represent those of their affiliated organizations, or those of the publisher, the editors and the reviewers. Any product that may be evaluated in this article, or claim that may be made by its manufacturer, is not guaranteed or endorsed by the publisher.
